# Task Uncertainty Can Account for Mixing and Switch Costs in Task-Switching

**DOI:** 10.1371/journal.pone.0131556

**Published:** 2015-06-24

**Authors:** Patrick S. Cooper, Paul M. Garrett, Jaime L. Rennie, Frini Karayanidis

**Affiliations:** 1 Functional Neuroimaging Laboratory, School of Psychology, University of Newcastle, Callaghan, Australia; 2 Priority Research Centre for Translational Neuroscience and Mental Health, University of Newcastle, Callaghan, Australia; Vrije Universiteit Brussel, BELGIUM

## Abstract

Cognitive control is required in situations that involve uncertainty or change, such as when resolving conflict, selecting responses and switching tasks. Recently, it has been suggested that cognitive control can be conceptualised as a mechanism which prioritises goal-relevant information to deal with uncertainty. This hypothesis has been supported using a paradigm that requires conflict resolution. In this study, we examine whether cognitive control during task switching is also consistent with this notion. We used information theory to quantify the level of uncertainty in different trial types during a cued task-switching paradigm. We test the hypothesis that differences in uncertainty between task repeat and task switch trials can account for typical behavioural effects in task-switching. Increasing uncertainty was associated with less efficient performance (i.e., slower and less accurate), particularly on switch trials and trials that afford little opportunity for advance preparation. Interestingly, both mixing and switch costs were associated with a common episodic control process. These results support the notion that cognitive control may be conceptualised as an information processor that serves to resolve uncertainty in the environment.

## Introduction

Cognitive control over thoughts and actions facilitates adaptation to our often uncertain environment, by enabling selective, goal-directed behaviours (e.g., [1–4]). Models of cognitive control posit a multi-process mechanism, subserved by complex frontal networks and activated under a range of contexts that require choice under conflict. These contexts can include detecting and resolving conflict [1], selecting responses, shifting between tasks, updating relevant rules [3,5], and learning novel associations [6].

Recently, Mackie, Van Dam & Fan (2013) [7] proposed that prioritisation of information processing may be the overarching mechanism by which cognitive control facilitates goal-directed behaviour. Given the limited capacity of the human attentional system, offering goal-relevant information privileged access to processing may contribute to efficient allocation of cognitive resources. Brain networks associated with cognitive control are consistent with privileged access through complementary systems. For instance, sustained control under demanding conditions is linked to sustained activity in medial frontal and insular regions, whereas transient control associated with rapidly changing goals is linked to activity in a distributed frontoparietal network [8–10]. Mackie and colleagues argue that when multiple actions are possible, a process of prioritisation may ensure that relevant information is processed expediently. As the number of action alternatives increases, so does the level of uncertainty, resulting in greater need for prioritisation. Many cognitive control paradigms involve high levels of uncertainty often as a result of conflict between multiple stimulus or response properties (e.g., distracting stimuli dimensions in Stroop tasks). Prioritising goal-relevant information in these tasks (e.g., word colour) may reduce uncertainty by limiting the influence of any additional conflicting features (i.e., word name).

From an information theory perspective, uncertainty is a measure of the information entropy that a given signal contains (c.f. [11]). More specifically, greater uncertainty arises from higher entropy or disorder within a signal. The level of entropy in a signal is typically measured in bits and is proportional to the number of possible states the signal could exist in. This is formalised in Eq 1:

$$\text{H}\left(X\right)=-\sum_{i=1}^{n}p_{\left(xi\right)}log_{2}p_{\left(xi\right)}$$ (1)

Here, the entropy (*Η*) of *X* is equal to the sum of the probability mass functions (*p(*_*Xi*_ *)log*_*2*_ *p(*_*xi*_ *))* for each possible value of *X*. As an example, consider two possible sets as below:

{xxxxx}{yyxyy}

In the first set, there is certainty that element *x* will be selected at random (i.e., probability of x being selected is 1) and so the entropy of *x* is zero. In the second set, the chance that *x* will be chosen randomly is only 0.2, giving *x* an entropy value of 2.32 bits. Therefore, in the first set, as the probability of selecting *x* is high and entropy is low, choosing *x* requires little information processing. However, in the second set, the probability of selecting *x* is low and entropy is high. Hence, selecting *x* requires an additional computational load to prioritise processing of relevant information. Thus, the concept of entropy may be useful in quantifying the computational load required to bring the system to the desired end-state.

Information entropy can be applied to typical interference paradigms (e.g., flanker tasks), by conceptualising interference as producing uncertainty and cognitive control as a computational mechanism that prioritises processing of information to resolve this uncertainty. For instance, in the example above, the two sets could be imagined as trials with congruent and incongruent flankers, respectively. The latter has greater entropy and hence cognitive control is required to prioritise processing of the central position in order to increase the probability that *x* will be selected. Fan, Guise, Liu, and Wang (2009) [12] used a Majority Function Task (MFT), where participants decide which direction the majority of arrows are facing, to quantify entropy as a function of arrow set size and congruence (the ratio of left vs. right pointing arrows). Reaction time increased linearly with congruence but not stimulus set size, so that high information entropy (i.e., small difference between the number of left and right pointing arrows) produced longer RTs irrespective of set size. This finding is consistent with the argument that increased entropy leads to more uncertainty and consequently the need for greater computational load to achieve the desired end-state. If cognitive control involves prioritising information to achieve task goals, the additional computational requirements for low congruence stimuli should manifest as longer RT (or processing time), as is seen in these data.

In this study, we examine whether this information-processing framework is also consistent with the variability in need for cognitive control within the context of task-switching paradigms. Dual modes of control models (e.g., [2]) argue that cognitive control can be activated both proactively, e.g., setting up the system in anticipation of a change in goal (proactive control) and reactively, e.g., to control interference and implement the goal (reactive control). Most interference paradigms (e.g., MFT, flanker) require reactive cognitive control. However, the task-switching paradigm requires both proactive and reactive cognitive control modes. Task-switching paradigms provide two indices of cognitive control. When alternating between two tasks in the same block of trials (i.e., mixed-task block), *switch* trials have slower RT and higher error rate than *repeat* trials (see [13–14]). This *switch cost* is at least partially attributed to a transient increase in cognitive control on switch trials in order to deal with the need to update goals and implement the new task set. Repeating the same task in a mixed-task block also has a cost compared to repeating in a single-task block. This *mixing cost* is at least partially attributed to a sustained increase in proactive cognitive control on mixed-task vs. single-task blocks. In the cued-trials variant of the task switching paradigm, a cue is presented prior to target onset and validly signals whether to switch or repeat task. Given a sufficiently long cue-target interval (CTI), proactive control can prepare the system to switch or repeat task in anticipation of target onset, resulting in a reduction in both switch cost and mixing cost. However, even with long CTIs, a residual cost remains, indicating that the need for reactive control to deal with target-driven interference processes (see [15,16]). Further, varying the information provided by the cue and the degree of conflict elicited by the target can manipulate the degree of task certainty at target onset. Thus, the cued-trials task-switching paradigm is well-suited to quantify the role of uncertainty on both proactive, preparatory control processes and target-driven, reactive processes.

**Fig 1:**
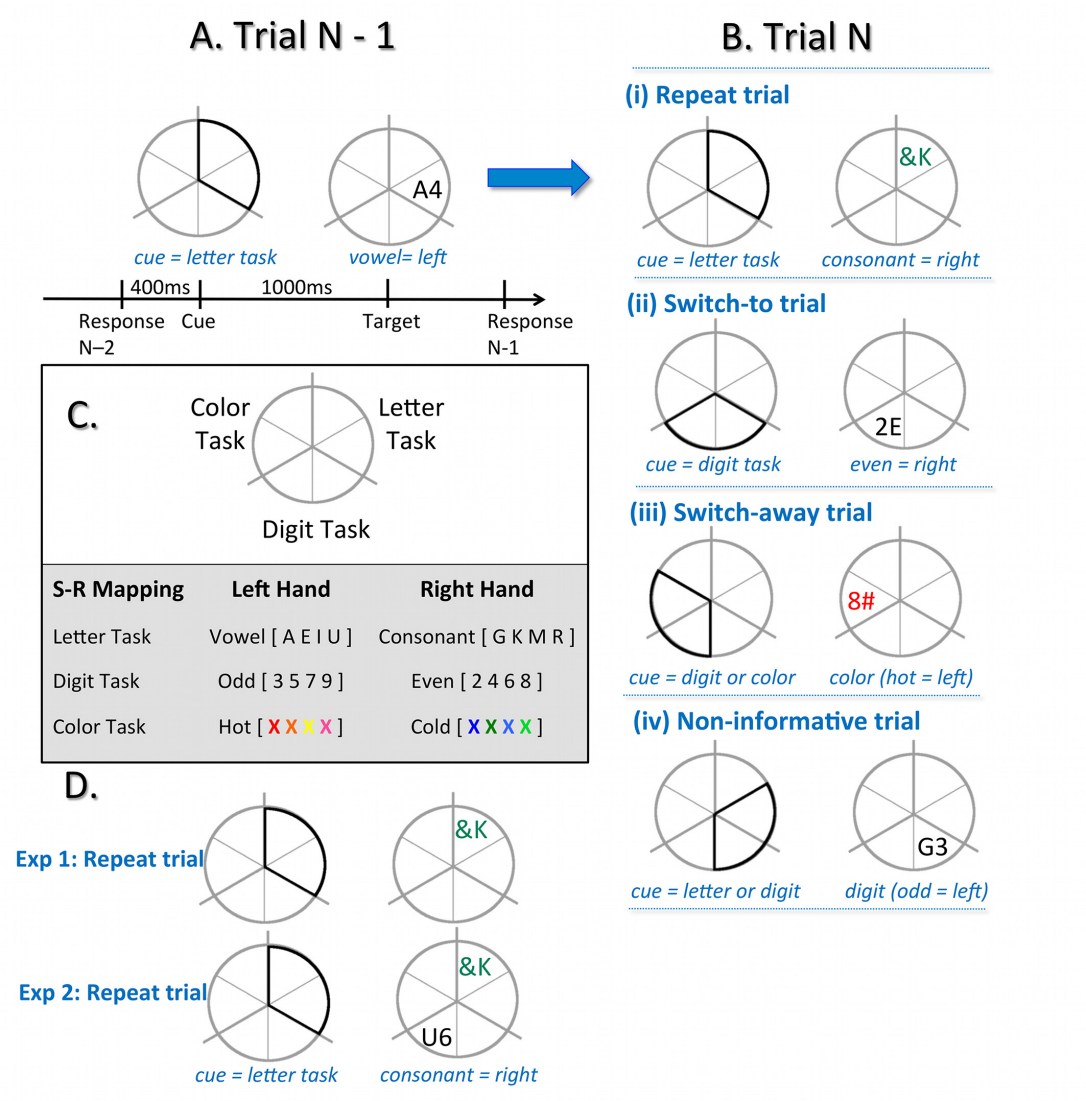
Cued-trial task switching paradigm. A) Timeline of a specific trial. Response-cue interval (RCI) and cue-target interval are fixed at 400 ms and 1000 ms, respectively. On each trial, the cue highlights two of the six segments of the circle and indicates that the target will appear in one of these two segments. In this instance, the cue covers both ‘letter task’ segments, and the participant can prepare to apply the ‘letter’ task rules on the upcoming target. When the target (e.g., A4) appears in a letter task segment, participants must respond to the task-relevant feature of the target (e.g., the letter A is a vowel, respond with left hand), and ignore the task-irrelevant feature of the target (e.g., the number 4). B) The progression from trial N-1 to trial N defines the trial type. i) If, having completed the letter task on trial N-1, the same segment is highlighted on trial N, it is a *repeat* trial and the participant will repeat the letter task. ii) If the cue highlights both segments of one of the other tasks, it is a *switch-to* trial. The target will appear in one of the two highlighted segments, and the participant can use the CTI to update the new task rules (e.g., digit task, in this example). iii) If the cue highlights adjoining segments of the two tasks not completed on trial N-1 (e.g., digit and color), it is a *switch-away* trial. The target is equally likely to appear in the digit and color segments and the participant can prepare to switch task (e.g., not repeat the letter task), but does not know which task to upload until the target appears. The position of the target indicates which task to complete. iv) If the cue highlights one segment from the task completed on trial N-1 (e.g., letter task) and one from another task (e.g., digit task), it is a *non-informative* trial. The target is equally likely to appear in the letter or the digit segment, and require either a repeat (*non-informative repeat*) or a switch (*non-informative switch*) in task. C) Each major segment of the wheel is consistently mapped to one of the three tasks: letter, digit and color. The table shows the eight exemplars used for each task and an example of stimulus-response mappings. D) Exemplar differences between a repeat trial for Experiment 1 and Experiment 2, whereby an additional bivalent distractor is presented at target onset in a non-cued section of the wheel during Experiment 2.

In the task-switching paradigm used here, the opportunity for proactive control was manipulated by varying the information value of the cue, whereas the need for reactive control was manipulated by varying level of interference at target onset (Fig 1; [17]). Experiment 1 manipulates the level of task certainty provided by the cue, so that different cues afford different opportunity for proactive control. Experiment 2 also manipulates the level of task certainty at target presentation, so that there is a greater need for reactive control than in Experiment 1. This resulted in various combinations of cue-level and target-level information entropy. The aim of this paper is to determine if variations in task uncertainty that impact on either the opportunity for proactive control or the need for reactive control contribute to behavioural costs and may therefore be suggestive of information prioritisation. We predicted that information entropy arising either because of differences in the level of preparation afforded by the cue (proactive control) or differences in task interference arising from the target (reactive control) would influence behavioural performance during task-switching. Specifically, if fully informative cues (i.e., cues that identify the upcoming task and allow advance task uploading) result in less entropy than partially informative or non-informative cues, they should be associated with less computational requirements and thus have faster RT and increased accuracy. Likewise, if ambiguous targets have high entropy (i.e., because they do not explicitly signal the relevant task), they will require higher computational allocation and result in slower RT and lower accuracy than unambiguous targets. These relationships between behavioural performance and information entropy would then account for *switch cost* and *mixing costs* in task-switching.

## Methods

### Participants

Experiment 1 included ninety-four community volunteers (mean age 23.76 ± 5.44 SD, range 17–35 years, 34 male) and Experiment 2 included 19 undergraduate students (mean age 19.42 ± 1.67 SD, range 18–24 years, 1 male). All participants reported no current psychiatric or neurological disorder.

### Ethics Statement

Both studies were approved by the University of Newcastle Human Research Ethics Committee (H-2012-0157), and complied with the Declaration of Helsinki. All participants provided informed written consent (approved by the University of Newcastle’s Human Research Committee) prior to participation in the respective studies and written parental consent was obtained for participants under 18 years of age.

### Task and Stimuli

For both experiments, participants were continuously presented a dark grey circle (5° visual angle) divided into six wedges. Pairs of adjacent wedges were marked with thicker lines to denote three task sections: letter, digit, and colour (Fig 1; see [17]). The target was a pair of characters consisting of combinations of a letter, a digit or a non-alphanumeric symbol and was presented either in grey or in colour. Each target (e.g., grey A4) consisted of three dimensions: one relevant to the currently cued task (e.g., the letter A mapped to left hand response), one selected randomly from one of the two alternative tasks and incongruently mapped with the relevant task (e.g., the digit 4 mapped to right hand response) and one that was neutral (e.g., letter and digit presented in grey that was not mapped to any response). In this way, targets always comprised identical levels of uncertainty regardless of trial type. The same target could not appear on successive trials.

One of four possible cue types preceded each target presented with equal probability in a pseudo-random sequence (i.e., same cue type was not repeated on more than four consecutive trials; Fig 1). The target always appeared in one of the two adjacent segments highlighted by the cue. *Repeat* cues indicated that the same task would be repeated. *Switch-to* cues indicated that the task would change and defined the new task. *Switch-away* cues indicated that the task would change, but did not specify which of the other two tasks would be relevant (i.e., the cue overlapped two segments mapped to tasks that were not relevant on the previous trial). *Non-informative* cues indicated that a switch or a repeat trial was equally likely (i.e., the cue overlapped two segments, one mapped to the previously relevant task and the other to a task that was not relevant on the previous trial). For both *switch-away* and *non-informative* cues, only the location of the target defined which task would be performed. Non-informative cues resulted equiprobably in a switch or a repeat trial.

Experiment 2 used an identical task, but with greater interference at target onset. As shown in Fig 1D, the target contained both the imperative stimulus (a bivalent stimulus which was relevant to the cued task and that required a response) and a distractor (a bivalent stimulus which was not relevant to the cued task and needed to be ignored). The distractor appeared in one of the non-cued task areas and comprised an additional pair of characters. The task-relevant feature of the distractor (e.g., the digit 4 if the distractor was in the digit segment) corresponded to the opposite hand of response to the target feature (e.g. the letter A in the letter segment, see Fig 1D). Hence, Experiment 2 had the same variation in entropy across trial types for the imperative stimulus as Experiment 1, but greater entropy at target onset as a result of the distractor.

### Procedure

Participants received training on both single-task and mixed-task blocks (a total of 1320 across both training sessions) in order to minimise learning effects on performance in the experimental session. The first training session was no longer than 14 days prior to the experimental session, and the second immediately preceded the experimental session. In Experiment 1, the experimental session consisted of ten mixed-task blocks (72 trials, plus five warm-up trials, per block) and three single-task blocks (48 trials, plus five warm-up trials, per block; one block per task). The single-task blocks were presented successively in a fixed order (e.g., letter, digit, colour) and were positioned randomly among the 10 blocks of the mixed-task sequence.

Each trial began with a cue that was replaced by the target (CTI 1000 ms). The target remained on screen until a response was issued or 5000 ms had elapsed. Errors were followed by a feedback tone. After each block, RT and accuracy feedback were provided and participants were encouraged to use this to maximise performance. A longer break was provided mid-way through testing to minimize fatigue. In Experiment 2, participants performed twelve mixed-task and three single-task blocks.

### Data Analyses

Warm-up trials and trials with RT less than 200 ms or more than three standard deviations above a participant’s mean RT were excluded from analyses. Following Fan et al. (2009) [12], we analysed RT and accuracy separately for Experiments 1 and 2. Note, task efficiency (i.e., accuracy/RT (in seconds)) produced results consistent with our other behavioural measures and with those of [12]. Behavioural data were extracted using MATLAB 2011b (The Mathworks Inc.) and statistical analyses undertaken in SPSS 21 (IBM). Data were analysed using a one-way repeated measures ANOVA with 6 levels of trial type and Greenhouse-Geisser corrections. To examine differences between switch and repeat trials as well as the use of informative cues, we performed the following planned contrasts on behavioural data: single-block-repeat vs. mixed-repeat, mixed-repeat vs. switch-to, mixed-repeat vs. non-informative repeat, switch-to vs. switch-away, switch-to vs. non-informative switch, switch-away vs. non-informative switch and non-informative repeat vs. non-informative switch. Bonferroni corrections were applied to these planned comparisons (.05/7 = *p* < .007).

### Analysis of task switching mental operation algorithms

To determine if differences in entropy during cued-trials task switching could account for differences in performance, we computed the information entropy for each trial type in both experiments. We adopted the algorithms proposed by [3] that quantify information entropy associated with cognitive control (Eq 2)

H(a)=I(s,a)+Q(a|s) (2)

where the total amount of information required for selecting an action *H(a)* is equal to the sum of the information entropy associated with sensorimotor processes *I(s*,*a)*, (*s*, stimulus and *a*, action) and cognitive control *Q*(*a*|*s*). These sensorimotor processes correspond to the amount of information that is exchanged between a stimulus (s) and the action (a). In the task-switching paradigm, the well-mapped information corresponding to target features and the appropriate response to be executed can be considered using sensorimotor control processes. Further, within cognitive control, Koechlin and Summerfield distinguish between information associated with contextual control (i.e., a context signal like a cue) and that associated with episodic control (i.e., previous events) as shown in Eq 3:

Q(a|s)=I(c,a|s)+Q(a|s,c) (3)

where contextual control *I(c*, *a|s)* measures the total amount of entropy conveyed by contextual signals *c* independent of *s*; and episodic control *Q(a|s*, *c)* measures the remaining entropy associated with past events independent of contextual signals. In task-switching, contextual control is captured by cues that provide information about the upcoming task whereas episodic control is other information provided in addition to these cues. For instance, in task-switching, the previous trial provides additional information beyond the cue to influence performance. That is, a repeat trial is defined not only by the contextual cue (e.g., a letter task) but by the information carried over by the previous trial (i.e., a letter task was just performed).

### Application of algorithm to other task switching studies

In order to examine the generality of the model, we applied the above algorithm to other studies that use variants of the cued-trials task switching paradigms. We searched the literature using PUBMED, ScienceDirect, PsychInfo, PsychArticle, PsychExtra and Google Scholar databases with terms “TASK SWITCHING”, “TASK SWITCHING AND CUE” and “SWITCH COST AND CUE” to find other cued-trials task switching paradigms published between January 2000 and December 2013. Studies were included if: 1) the target was preceded by a cue, 2) trials were not presented in a predictable order (i.e., either random or pseudorandom presentation), 3) the study reported mean RTs and 4) the paradigm did not include additional cognitive manipulations (e.g., a cued-trials task switching paradigm which included no-go trials). These criteria allowed us to compute uncertainty for switch and repeat trial types using the mental algorithm presented here. We report the regression values and study characteristics. Note that, for simplicity of analysis given a broad range of CTIs, a CTI of less than 200 ms was classified as a non-informative condition (nb. this applied to the following studies: [18–24]). Additionally, for [25] we merged the data from the younger age groups into a single young age group.

## Results

### Behavioural Results

For Experiment 1, there were significant main effects of trial type for mean RT and accuracy (*F*(5,465) = 307.53, *p* < 0.001, *F*(5,465) = 16.42, *p* < .001, respectively). As shown in Table 1, there was a significant difference between repeat trials on single-task and mixed-task blocks on RT (*t*(93) = -12.31, *p* < .001) but not accuracy. Mixed-repeat trials were faster than both switch-to (RT; *t*(93) = -11.32, *p* < .001) and non-informative repeat trials (RT; *t*(93) = -16.66, *p* < .001). Fully informative switch trials (i.e., switch-to) were faster than partially informative switch trials (i.e., switch-away; RT; *t*(93) = -21.4, *p* < .001) and non-informative switch trials (RT; *t*(93) = -18.31, *p* < .001). There were no significant differences between switch-away and non-informative switch trials for RT, but the former were more accurate (*t*(93) = 5.76, *p* < .001).

**Table 1:** Task switching RT (ms) and accuracy (%) with standard error (SE), for all conditions in the standard task switching paradigm.

Block	Condition	RT ± SE (ms)	Accuracy ± SE (%)
**Single**	Repeat	570 ± 8.5	98.9 ± 0.1
**Mixed**	Repeat	673 ± 14.48	97.9± 0.2
	Switch-to	796 ± 20. 93	96.6 ±0.2
	Switch-away	918 ± 20.14	96.0 ± 0.2
	Non-informative repeat	782 ± 13.36	97.2 ±0.2
	Non-informative switch	923 ± 20.46	95.3 ±0.3

For Experiment 2, there was a significant main effect of trial type on RT (*F*(5,90) = 88.93, *p* < 0.001), but not on accuracy (see Table 2). Despite having a much smaller sample size than Experiment 1, Experiment 2 produced highly compatible RT outcomes. RT was faster for repeat trials in single-task than mixed-task blocks (*t*(18) = -5.73, *p* < .001). Mixed-repeat trials were faster than both switch-to (*t*(18) = -6.08, *p* < .001) and non-informative repeat trials (*t*(18) = -8.59, *p* < .001). Fully informative switch trials (i.e., switch-to) were also faster than partially informative switch (i.e., switch-away; RT; *t*(18) = -10.69, *p* < .001) and non-informative switch (*t*(18) = -11. 43, *p* < .001) trials. Finally, non-informative repeat trials were faster than non-informative switch trials (*t*(18) = -5.03, *p* < .001).

**Table 2:** Task switching RT (ms) and accuracy (%) with standard error (SE), for all conditions in the distractor task switching paradigm.

Block	Condition	RT ± SE (ms)	Accuracy ± SE (%)
**Single**	Repeat	597 ± 19	97.4 ± 0.3
**Mixed**	Repeat	709 ± 30.8	97.1 ± 0.2
	Switch-to	837 ± 47.3	96.9 ± 0.4
	Switch-away	1022 ± 52.2	96.8 ± 0.4
	Non-informative repeat	889 ± 41.4	97.3 ± 0.3
	Non-informative switch	1004 ± 48	96.6 ± 0.4

### Information Entropy

Entropy was determined for each trial type across both experiments using Koechlin & Summerfield’s (2007) [3] cognitive control algorithms. Table 3 shows the implementation of these algorithms and the resulting information entropy for each condition. Following [12], each component of the control algorithm was provided a representative value to reflect level of input for each of the three factors considered by the algorithm (i.e., sensorimotor, contextual and episodic control). For example, for repeat trials in single-task blocks in Experiment 1, there is one level of input into the contextual stage (i.e., one task is cued) and two levels of sensorimotor input (i.e., one task-relevant feature of the target to process, and one response hand based on that target feature). In comparison, for repeat trials in the single-block in Experiment 2, there are four levels of input for the sensorimotor component, as there were two combinations possible depending on appropriate use of the contextual cue (i.e., two inputs associated with the target and two with the distractor). Note that these values represent the amount of input rather than a specific parameter that is extracted for the algorithm, so that an input of zero does not represent a complete lack of input. As such, in the single-task block, episodic demands are limited as the same task is repeated and thus task-set maintenance across a block can be represented by a zero value for episodic control.

**Table 3:** Mental operation algorithms and equivalent entropy values (bits) for the standard and distractor task switching paradigms.

Block	Condition	Sensorimotor Control	Cognitive Control		Bits (log_2_)
		Sensorimotor	Contextual	Episodic	
**Single**	SBR	2	1	0	1
	SBR-D	4	1	0	2
**Mixed**	MR	2	1	1	2
	MR-D	4	1	1	3
	ST	2	1	2	2.58
	ST-D	4	1	2	3.58
	SA	2	2	2	3
	SA-D	4	2	2	4
	NR	2	2	1	2.58
	NR-D	4	2	1	3.58
	NS	2	2	2	3
	NS-D	4	2	2	4

Note: SBR; Single Block Repeat, MR; Mix Repeat, ST; Switch To, SA; Switch Away, NR; Non-informative Repeat, NS; Non-informative Switch. Trials from Experiment 2 are denoted by-D (i.e., distractor).

As seen in Table 3, information entropy for each condition in Experiment 1 was smaller than the equivalent condition in Experiment 2 because in the latter paradigm, the distractor caused additional sensorimotor entropy. Moreover, within each experiment, *repeat* trials in *mixed*-*task* blocks had higher entropy than *repeat* trials in *single*-*task* blocks; *switch* trials had higher information entropy than *repeat* trials in mixed-task blocks and partially informative (*switch-away*) and *non-informative* trials had higher entropy than fully informative trials (*repeat* and *switch-to* trials).

**Fig 2:**
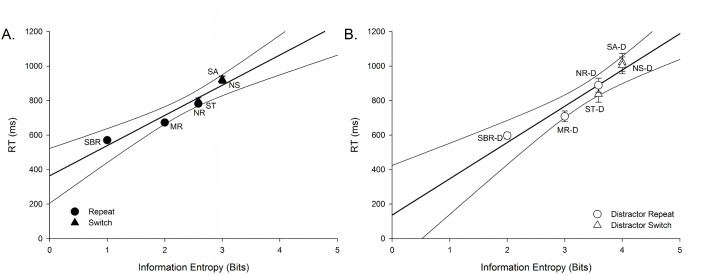
Reaction time as a function of information entropy for each trial type in A) the standard, and B) the distractor cued-trials task switching paradigms. Information processing requirements (in bits) were assumed to differ based on an increased entropy for mixed blocks in contrast to single blocks, with the addition of an extra degree of uncertainty for switching within a mixed-task block. The above algorithm strongly predicted mixing and switch costs for RT in both Experiment 1 (A: r^2^ = .934, p = .002) and Experiment 2 (B: r^2^ = .924, p = .002). SBR; single block repeat, MR; mixed repeat, ST; switch-to, SA; switch-away, NR; non-informative repeat, NS; non-informative switch. –D denotes distractor paradigm trials.

**Fig 3:**
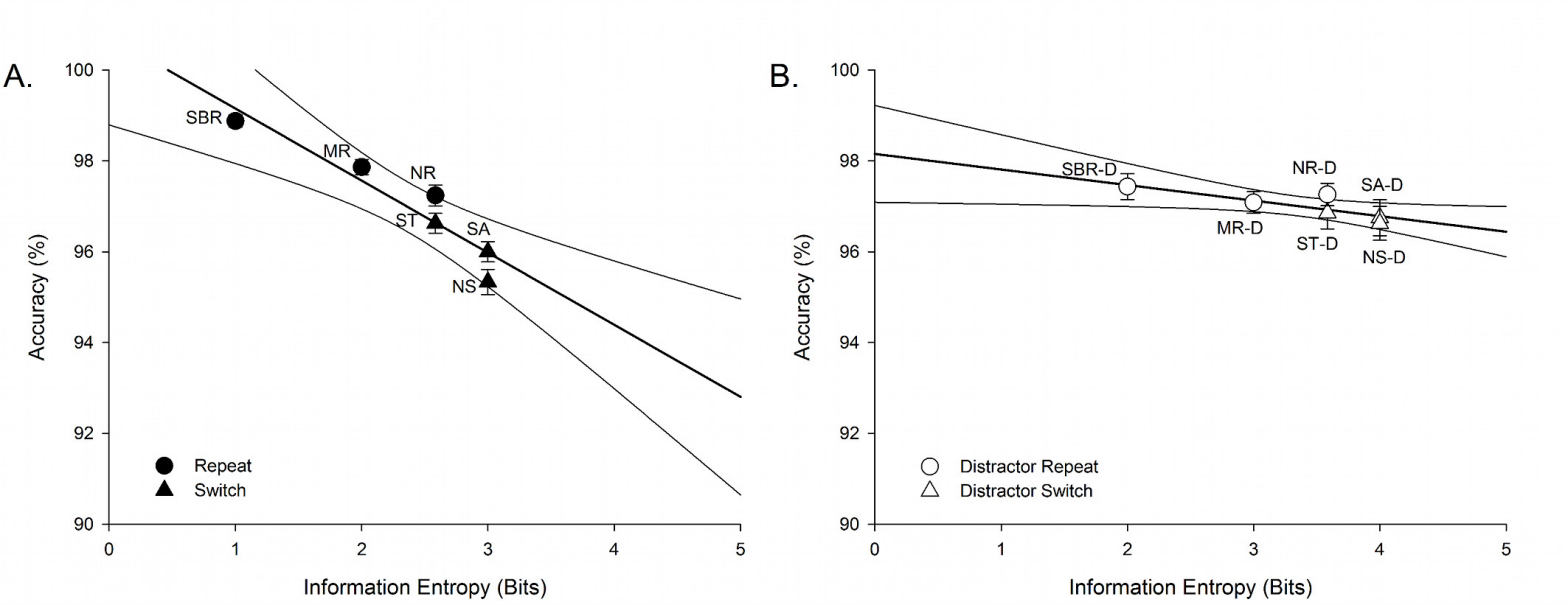
Accuracy as a function of information entropy for each trial type in A) the standard, and B) the distractor cued-trials task switching paradigms. As with RT, information entropy (bits) strongly predicted behavioural performance for both Experiment 1 (A: r^2^ = .886, p = .005) and Experiment 2 (B: r^2^ = .701, p = .038). SBR; single block repeat, MR; mixed repeat, ST; switch-to, SA; switch-away, NR; non-informative repeat, NS; non-informative switch. –D denotes distractor paradigm.

We performed linear regression for the average behavioural performance within each trial type and information entropy to determine if higher uncertainty affected behavioural performance in a manner consistent with greater computational requirements (or cognitive control; i.e., slower RT and reduced accuracy). For both experiments, increasing entropy was associated with longer RT (Experiment 1: Fig 2A, r^2^ = .934, p = .002; Experiment 2: Fig 2B, r^2^ = .924, p = .002) and lower accuracy (Experiment 1: Fig 3A; r^2^ = .886, p = .005; Experiment 2: Fig 3B, r^2^ = .701, p = .038). The pattern of outcomes was very similar across experiments, but the relationship between entropy and accuracy was weaker in Experiment 2. Given the large differences in sample sizes between experiments (i.e., Experiment 1, n = 94; Experiment 2, n = 19), we also performed these analyses with a smaller subset of participants from Experiment 1, matched for age with Experiment 2 (n = 19; mean age 19.68 ± 1.8; 10 female, 9 male). Using this smaller subset, we found comparable results with the larger sample, with a significant relationship between entropy and RT (r^2^ = .964, p < .001) and entropy and accuracy (r^2^ = .885, p = .005).

Notably, in both experiments, switch-to trials had higher RT, lower accuracy and higher information entropy than mixed-repeat trials. Likewise, mixed-repeat trials had longer RTs, lower accuracy and higher information entropy than single-block-repeat trials. Therefore, both switch cost (switch-to vs. mixed-repeat trials) and mixing cost (mixed-repeat vs. single-block-repeat trials) were associated with differences in information entropy between conditions.

### Does this algorithm fit task-switching performance in other studies?

As shown in Figs 2 and 3, information entropy very consistently predicted behavioural performance in Experiments 1 and 2. Next, we examined whether the above algorithm can fit the results of other studies using variants of the cued-trials task-switching paradigm. As regression analyses indicated that the most robust association between behavioural performance and entropy was for RT, we restricted analyses to RT only. Table 4 lists RT from 20 experiments reported in 12 studies using a cued-trials task-switching paradigm, with a brief description of the study characteristics.

**Table 4:** Modelling of task switching mental algorithm to cued-task switching paradigms.

Experiment	Switch Classification Types	Subgroup	N	Mean Age (± SD)	Condition (CTI)	RT (ms)	Entropy (bits)	R^2^ adj	SE
**Braver et al. (2003) [26]**	Judgement: (artificial/natural)		13	21 (range: 19–26)	SBR (2500)	969	1	.971	13.68
	Magnitude: (large/small)				MR	1053	2		
					S	1129	2.58		
**Braverman & Merian (2010) [27]**	Spatial location: (up/down vs. left/right)	Experiment 1	16	-				.64	51.42
		Low conflict			MR (1000)	538	2		
					S	550	2.58		
					NR (100)	655	2.58		
					NS	706	3		
		High conflict			MR (1000)	587	2.58		
					S	608	3		
					NR (100)	721	3		
					NS	774	3.32		
		Experiment 2						.524	75.9
		Low conflict			MR (1000)	435	2		
					S	452	2.58		
					NR (100)	577	2.58		
					NS	642	3		
		High conflict			MR (1000)	524	2.58		
					S	485	3		
					NR (100)	696	3		
					NS	719	3.32		
		Experiment 3						.441	66.04
		Low conflict			MR (1000)	424	2		
					S	426	2.58		
					NR (100)	552	2.58		
					NS	562	3		
**Goffaux et al. (2006) [28]**	Judgement: (non/living)		20	24.5 (3.4)	SBR (1180)	550	1	.991	14.03
	Size: (large/small)				MR	748	2		
	Breadth: (wide/narrow)				S	837	2.58		
**Grange & Houghton (2010) [18]**	Iconic: (shape)		32	-	MR (900)	529	2	.525	51.41
	Word: (word)				S	544	2.58		
					NR (100)	640	2.58		
					NS	683	3		
**Jost et al. (2008) [29]**	Colour: (blue, red, green or yellow)		16	22	SBR (1000)	703	1	.666	76.4
	Shape: (circle, triangle, square or cross)				MR	779	2		
					S	813	2.58		
					NR (200)	906	2.58		
					NS	1047	3		
**Kray (2006) [19]**	Category: (animal/not)	Young adults	16	21 (2.4)				.884	26.75
	Syllable: (one/two)	CTI 400			SBR (400)	563	1		
					MR	707	2		
					S	803	2.58		
		CTI 750			SBR (750)	599	1		
					MR	699	2		
					S	740	2.58		
		CTI 2300			SBR (2300)	632	1		
					MR	727	2		
					S	757	2.58		
**Meriran et al. (2000) [20]**	Spatial location: (up/down vs. left/right)	Congruent	10	-	MR (232)	571	2	.743	28.52
					S	653	2.58		
					MR (432)	570	2		
					S	608	2.58		
					MR (1032)	533	2		
					S	592	2.58		
					MR (3032)	570	2		
					S	617	2.58		
					NR (132)	619	2.58		
					NS (132)	735	3		
		Incongruent	10	-	MR (232)	613	2	.663	33.07
					S	736	2.58		
					MR (432)	626	2		
					S	684	2.58		
					MR (1032)	606	2		
					S	651	2.58		
					MR (3032)	627	2		
					S	637	2.58		
					NR (132)	668	2.58		
					NS (132)	782	3		
**Merian & Daichman (2005) [21]**	Spatial location: (up/down vs. left/right)	Unspeeded	12	-	MR (600)	523	2	.41	46.59
					S	517	2.58		
					MR (2500)	500	2		
					NR (100)	577	2.58		
					NS	656	3		
		Speeded			MR (600)	471	2	.443	42.91
					S	470	2.58		
					MR (2500)	471	2		
					NR (100)	474	2.58		
					NS	609	3		
**Nessle et al. (2012) [22]**	Digit magnitude: (greater/less than 5)	Infrequent switch	16	25.4 (range: 18–30)	SBR (1300)	496	1	.797	62.81
	Digit parity: (odd/even)				MR	602	2		
					S	663	2.58		
					NR	715	2.58		
					NS	872	3		
		Frequent switch			SBR (1300)	496	1	.861	48.24
					MR	611	2		
					S	665	2.58		
					NR	745	2.58		
					NS	836	3		
**Ruge et al. (2005) [23]**	Spatial location: (up/down vs. left/right)		18	25.5 (range: 21–35)	MR (2000)	554	2	.653	38.32
					S	573	2.58		
					NR (100)	616	2.58		
					NS	700	3		
**Terry & Sliwinski (2012) [24]**	Digit magnitude: (greater/less than 5)	Young	26	18.4 (1.1)	SBR (100)	599	1	.969	54.18
	Digit parity: (odd/even)				NR	1010	2.58		
					NS	1206	3		
		Old	25	80.3 (5.4)	SBR (100)	790	1	.994	38.66
					NR	1523	2.58		
					NS	1778	3		
**Whitson et al. (2012) [25]**	Letter: (vowel/consonant)	Young	45	29 (range 18–40)	SBR (1000)	631	1	.821	73.6
	Digit: (odd/even)				MR	748	2		
					S	848	2.58		
					NR (150)	929	2.58		
					NS	1089	3		

Entropy value was determined using the algorithm developed in this study and the final column shows the adjusted R^2^ values for each experiment. Information entropy was a good predictor of RT in all experiments. The average adjusted R^2^ value was .733 (+/-.19 SD); 50% of studies had an R^2^ value above 0.75 and only three experiments had a value below 0.5 (but still greater than 0.4). Therefore, just as information entropy was able to predict behavioural performance across our two task-switching experiments the results from many other task-switching studies can also be interpreted within the information entropy framework employed here.

## Discussion

The current study aimed to determine if information entropy could account for behavioural costs during task-switching under conditions that emphasise proactive control or both proactive and reactive control. Increasing uncertainty (quantified as information entropy in bits) was associated with longer RT and reduced accuracy, consistent with increased computational demands on the control system. Furthermore, *switch cost* and *mixing cost*, the specific indices of cognitive control implementation in task-switching paradigms, varied as a function of systematic differences in information entropy between trial types. This increased entropy for switch trials was evident in both the task-switching paradigms used here, as well as in other studies using cued-trials task switching. In conclusion, our results show that information prioritisation to resolve task uncertainty can account for task-switching performance and thereby support the notion of cognitive control as a process of information prioritisation (see [7,30]).

Information theory predicts that increased entropy or uncertainty results in greater need for information processing which results in longer computation times (e.g., longer RT [31]) and/or greater energy requirements (e.g., greater brain activation, [32]). In this study, higher levels of entropy resulted in an increase in computation times (i.e., longer RT), consistent with the notion of an active information processor being increasingly engaged with higher uncertainty. The current findings are consistent with recent studies suggesting a relationship between information entropy and need for cognitive control ([7,12,33,34]). To our knowledge, this is the first study to show that information entropy can explain behaviour under both proactive and reactive modes of cognitive control.

### Episodic control influences on task-switching

A comparison between information content of subcomponents of cognitive control and task-switching performance is possible using Koechlin and Summerfield’s (2007) [3] control algorithm. While regression analyses showed a linear increase in RT (and decrease in accuracy) with increasing information entropy across both experiments, the amount of information content in subcomponents of control also varied between trial types. For instance, *contextual control* differences (i.e., opportunity to prepare for a task using the cue) were apparent between conditions with different RTs (e.g., mixed-repeat (informative) trials had less contextual entropy and were faster than non-informative repeat trials; Fig 2A), suggesting that information content during the preparation interval can influence performance. Likewise, in agreement with Hick-Hyman Law [35], performance in Experiment 1 was generally faster than in Experiment 2, in which additional *sensorimotor* processing was required at target onset to overcome distractors.

Interestingly, behavioural switch costs (i.e., switch—repeat) had corresponding differences in information entropy associated with the need for *episodic control*. By definition, task repetition requires control processes associated with maintaining the previous task set, and these cannot be captured by contextual or sensorimotor control. Switching between tasks requires additional processes that are dependent on episodic control (e.g., disengagement of previous task set). Thus, entropy differences between repeat and switch trials can be driven by episodic control and may play a key role in the behavioural switch cost. Likewise, mixing costs may arise from episodic control differences between repeating in single task and mixed blocks. Episodic control demands are lower when repeating in a homogenous block, as the high level of predictability should lower the need to continuously maintain or manipulate previous task set characteristics on the current trial. Therefore, differences in the need for episodic control between repeat trials in mixed-task and single-task blocks, as well as between switch and repeat trials in mixed-task blocks, may underscore a common process to both the mixing and switch cost.

Previous work has shown that *mixing* and *switch* costs are not independent processes (e.g., see [36, 37]). For example, inter-trial interference in mixed-task blocks may account for the magnitude of both switch cost and mixing cost (e.g., [14]). Likewise, working memory processes, such as maintenance of multiple task sets or trial-by-trial retrieval of task rules [38,39], differ between single-task and mixed-task blocks. Episodic control information entropy is compatible with these previous accounts of switch and mixing costs. For instance, task-set carryover effects, like passive task-set decay (e.g., [20,40]) can result in differential demand for an episodic control process in single-task and mixed-task blocks. This difference can be captured through information entropy of episodic control, so that repeating in a mixed-task block has higher entropy than in a single-task block. Similarly, within mixed-task blocks, repeat trials require only maintenance of the task-set whereas switch trials require the previous task-set to be disengaged and the current one uploaded. These differences in trial-by-trial processes can also be captured by episodic control entropy. Thus entropy differences in episodic control processes may fit well within previous cognitive accounts of task-switching dynamics and offer a novel insights into cognitive control.

### Information entropy captures situational factors influencing control

The current study provides further evidence that need for cognitive control may relate to situational differences in information entropy. Increasing entropy has previously been shown to arise in situations that models of cognitive control often refer to as *conflict* [1], i.e., situations that require a choice in the presence of multiple alternatives. Typically, these instances of conflict are conceptualised as competition between two or more possible goals, wherein the control system is employed to resolve this conflict. Information theory models of cognitive control can account for these instances of conflict (e.g., the bivalency of the targets in the current study or stimulus features in [7,12]) by arguing that conflict results in increased entropy, which can be overcome by prioritisation of resources. In the present study, we show that entropy differences also exist in contexts that are not typically associated with conflict, such as using contextual cues to prepare for a task. This novel insight extends the relevance of entropy models of control beyond conflict-based contexts [1].

Increased entropy arising from both contextual cueing and conflict fits well with models of cognitive control that invoke dual modes of control (DMC; [2]), i.e., proactive and reactive processes, in the flexible adjustment of behaviour. Braver [2] suggests that situational factors can change the balance and prioritise the use of one control mode over the other. In the current context, differences in performance between trial types result from differential use of proactive vs. reactive control processes. For instance, the performance difference between informative repeat trials (i.e., *mixed-repeat*) and *non-informative repeat* trials can be explained by a difference in the opportunity to use preparatory control processes and was captured well by a difference in information entropy during contextual control. By quantifying the levels of entropy during proactive (e.g., contextual) and reactive (e.g., sensorimotor) control, information theory mechanisms of control are well aligned within DMC frameworks.

However, the fact that partially informative (*switch-away*) and *non-informative switch* trials had the same entropy and resulted in similar level of performance, is seemingly at odds with DMC: the opportunity for partial preparation for switch-away trials should have resulted in less entropy and better performance. In previous work using formal models of decision-making, we have shown that, in line with DMC, *switch-away* and *non-informative switch* trials do in fact differ in decision parameters that contribute to RT. Specifically, switch-away trials had a higher response threshold and lower non-decision time [17] than non-informative switch trials, consistent with proactive control increasing cautiousness but reducing non-decision processes. Yet, these trial types did not differ in the rate of evidence accumulation.

Taken together, proactive and reactive situational factors that contribute to the use of cognitive control may make different contributions to overall information entropy and resulting performance. While research into information entropy accounts of cognitive control have focused on the end point of information prioritisation (i.e., RT or accuracy, [7,12]), it is possible that different decision and non-decision processes that contribute to overall task performance (i.e., RT and accuracy) are differentially indexed by entropy. Future work needs to examine these interrelationships.

## Conclusion

Cognitive control is important in a vast range of situations where habitual responding is insufficient to meet goals. Recently, it has been proposed that cognitive control may be conceptualised as a process of prioritisation of information in order to resolve uncertainty (e.g., [7,30]). Here, we show that uncertainty can account for differences in behavioural performance during task-switching and may provide insight into factors that influence mixing and switch costs.
